# Serum Albumin Nanoparticles: Problems and Prospects

**DOI:** 10.3390/polym13213759

**Published:** 2021-10-30

**Authors:** Viktória Hornok

**Affiliations:** 1 Department of Physical Chemistry and Materials Science, University of Szeged, Rerrich B. Square 1, H-6720 Szeged, Hungary; vhornok@chem.u-szeged.hu; Tel.: +36-62-544211; 2 MTA Premium Post Doctoral Research Program, Rerrich B. Square 1, H-6720 Szeged, Hungary

**Keywords:** serum albumin, nanoparticles, drug delivery, protein carriers

## Abstract

The present paper aims to summarize the results regarding serum albumin-based nanoparticles (NPs) for drug delivery purposes. In particular, it focuses on the relationship between their preparation techniques and synthesis parameters, as well as their successful clinical application. In spite of the huge amount of consumed material and immaterial sources and promising possibilities, products made from different types of albumin NPs, with the exception of a few, still have not been invented. In the present paper, promising applications of serum albumin nanoparticles (SANPs) for different biomedical purposes, such as carriers, delivery systems and contrast agents, are also discussed. The most frequent utilization of the NPs for certain diseases, i.e., cancer therapy, and future prospects are also detailed in this study.

## 1. Introduction

With the present need for improving health properties through the application of modern, smart, next-generation drug delivery systems (DDS), the application of nanoparticles (NPs) to free drugs is advantageous for several reasons [1,2,3]. With the application of drug delivery systems, poorly soluble drugs can be solubilized to accommodate therapeutic cargoes within their particle cores [4]. When these drugs are protected by the carrier and their degradation is prevented, their half-life can be appropriately adjusted and the release of their properties can be sustained [5] or tuned according to the proposed application. Thus, this will ensure the required administration and reduce side effects [6,7]. From the carrier’s point of view, the next-generation half-life extension and targeted drug-delivery technologies in general should be simple in design, biocompatible, biodegradable and nonimmunogenic. Moreover, they should be targeted or accumulated in the target tissue or body compartment [8,9]. The different potential DDS can be distinguished by their size, shape, composition etc. They can be formed using plenty of materials involving natural or synthetic polymers like polymeric nanoparticles [10,11], dendrimers, micelles [12] or lipids (liposomes) [13,14], and viruses (viral NPs). Albumin, as an exogenous or endogenous carrier protein for treating various diseases—primarily cancer [15], rheumatoid arthritis, diabetes and hepatitis—has demonstrated its potential in the form of products and numerous clinical trials [16]. Human serum albumin (HSA) can be the possible solution to this unmet challenge [17,18]. The albumin is a family of globular proteins, the most common of which are the serum albumins [19]. Abraxane^®^ is a drug formulation based on SANPs that garnered more than 600 million dollars in sales in 2012 alone [20] and saw 52% sales growth in 2013 [21]. Furthermore, Abraxane^®^ is considered by experts to be one of the main approaches to treating all types of cancer in the near future [22,23]. Albumin-based DDS, ranging from SANPs, albumin fusion proteins, prodrugs and peptide derivatives that bind covalently to albumin as well as physically binding antibody fragments and therapeutically active peptides, are in advanced clinical trials or approved products [24]. Due to its role in organisms in which it acts as a carrier of fatty acids and many molecules (including vitamins (C, D), folate, and steroid hormones) and minerals (such as copper, zinc or calcium, etc.), albumin plays an important role in stabilizing the blood pH via its buffering action and is solely responsible for more than 80% of plasma osmotic pressure. Its advantageous properties include the possibility of drug encapsulation due to its binding sites for various ligands, i.e., Sudlow’s sites. However, in spite of its obvious potential and the numerous scientific reports thereon, the clinical application of albumin-based therapy is still limited. To date, only a few products are present in clinical applications [25,26]. The aim of this review is to summarize the present possibilities and provide ideas about the potential future utilization technologies.

## 2. Role of Albumin in Humans and Its Potential

Over the past few decades, albumin has emerged as a powerful macromolecular carrier in medical therapeutic and diagnostic [27] applications [20]. Its role in binding to the neonatal Fc receptor (FcRn) was investigated by Anderson et al., and its pH-dependent manner of binding was also clarified [28,29]. The medical application of serum albumins can be regarded favorably for at least three main reasons. Firstly, its molecular weight lies above the renal threshold with a long circulation time; thus, it can be accumulated in inflamed and malignant tissues. Secondly, the Gp60 receptor on endothelial cells is responsible for the transcytosis of albumin and aids in transporting this protein into the tumor against the efflux induced by the interstitial fluid pressure of solid tumors. Additionally, the FcRn binds IgGs but is also responsible for the long half-life of the protein in the body. Thirdly, albumin’s high abundance and multiple binding sites promote improved pharmacokinetic properties of therapeutically active peptides or small-sized antibody moieties.

As albumin has its own role in the body as a blood component, it can count as a therapeutic agent on its own or can be used as a drug carrier system [30], diagnostic agent in the diagnosis of diseases such as tuberculosis and acquired immunodeficiency syndrome (AIDS) [31], and can also be used as a coating agent [32,33]. The interaction between drugs and albumins is still greatly investigated by researchers, even if their therapeutic relevance is questionable for most cases since the changes in plasma protein binding may influence individual plasma protein binding, which will not influence the clinical exposure of a patient to a drug [34,35]. Its role in drug delivery is shaped by the formation of the so-called protein corona on the NPs [36,37,38,39]. The extensive investigation of albumin in cancer therapy is due to the several factors that lead to its preferable accumulation in tumor cells [40,41]. For instance, neoplasm, a disease category including malignant cancer, is a principle cause of mortality worldwide and its most common and accepted treatment includes radiation therapy, chemotherapy and surgery. The latter is considered to be the first most successful treatment for various types of solid tumors [42].

### 2.1. Source of Albumin

**Figure 1:**
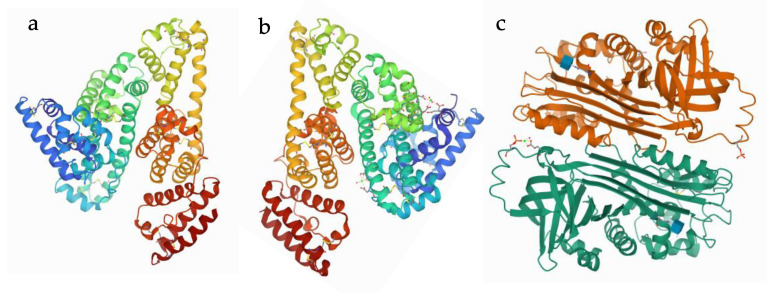
The 3D structure of albumins: (**a**) human serum albumin; (**b**) bovine serum albumin and (**c**) ovalbumin [49].

Albumin can be purchased from several commercially available sources (for commercial use), including vegetable, animal and human sources. The most commonly used albumins include egg-white (called ovalbumin), bovine serum albumin (BSA) and human serum albumin (HSA) (Figure 1) [43,44,45,46]. Among them, BSA is widely accepted and applied in research because of its low cost, and easy availability and purification [47]. However, milk, soy and legumes, among others, provide a new class of albumin base, recently providing the possibility for so-called green preparation [48]. HSA is used in studies involving humans to avoid any immunological responses and to eliminate any disadvantages of animal-based serum albumin, including diseases such as bovine spongiform encephalopathy [48]. Nevertheless, there has been a widespread application of ovalbumin in the food industry due to its ability to form foam and gel networks.

### 2.2. Structural Properties

The three-dimensional structure of human serum albumin is determined by X-ray crystallography, which proved that it presents in a characteristic heart-shape structure with three elliptical sub-domains: Domain I (residues 1–195), Domain II (residues 196–383) and Domain III (residues 384–585), with dimensions of 80 by 30 Å [50,51]. The serum albumin is a globular, single-chain protein, and its structure is determined by, and its high stability is attributed to, the internal formation of 17 disulfide bonds from 34 of the consisting 35 cysteine amino acids. The presence of a large number of charged amino acids such as lysine, arginine, glutamic acid and aspartic acid plays a variety of important biological roles [52,53]. The structural properties of albumin provide some other advantages in the production of NPs, such as the presence of numerous functional groups of the consisting amino acid, making it possible for the insertion of drugs of different character (positively charged, negatively charged, ambivalent, hydrophobic or hydrophilic). Due to this, the surface functionalization (that is, the appropriate modification to ensure conjugation for targeted delivery) is also relatively easy compared to other carriers [54].

### 2.3. Drug Encapsulation

Sudlow’s site I mainly binds the dicarboxylic acids and bulky heterocyclic molecules and Sudlow’s site II, or the indole-benzodiazepine site, has affinity towards the aromatic carboxylic acids [55]. The tag of the serum albumin family possesses remarkable water solubility and stability. Serum albumins are even moderately soluble in concentrated salt solutions, and are able to withstand temperatures of 60 °C for 10 h [53]. Due to the specific structure of albumins, the release of drugs is possible in the bloodstream. However, there are certain drawbacks, such as the necessity of, in some cases, toxic cross-linking to increase the stability of ABNPs to hinder swelling and dilution in vivo, since the fast introduction of drugs into water prior to reaching the target tissue in the body results in the fast outburst of the drugs.

## 3. Advantages and Disadvantages of Different Preparation Methods of SANPs

Among the several advantages of using albumin as a carrier, as a component of blood plasma with high abundance, its immunogenicity, biocompatibility and biodegradability are the most obvious ones. Moreover, due to its important physiological roles in the human body, high levels of albumin can be inserted into the organisms without or with low side effects.

**Figure 2:**
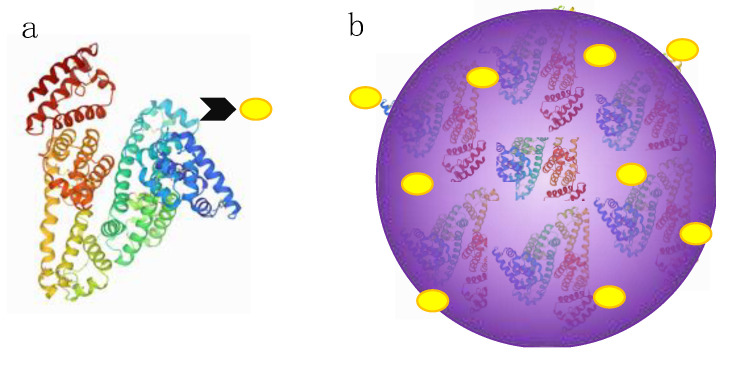
Simplified preparation methods of two different approaches to albumin-based drug delivery systems: (**a**) drug-conjugated albumin and (**b**) drug-containing NPs.

Basically, two approaches to producing albumin drug carrier systems can be observed: (i) the chemical coupling of drug molecules to single albumin molecules to form albumin–drug conjugates and (ii) the encapsulation of drugs in the NPs formed from several albumins via supramolecular forces within its multiple binding sites or adsorbed on its surface (Figure 2) [56].

**Figure 3:**
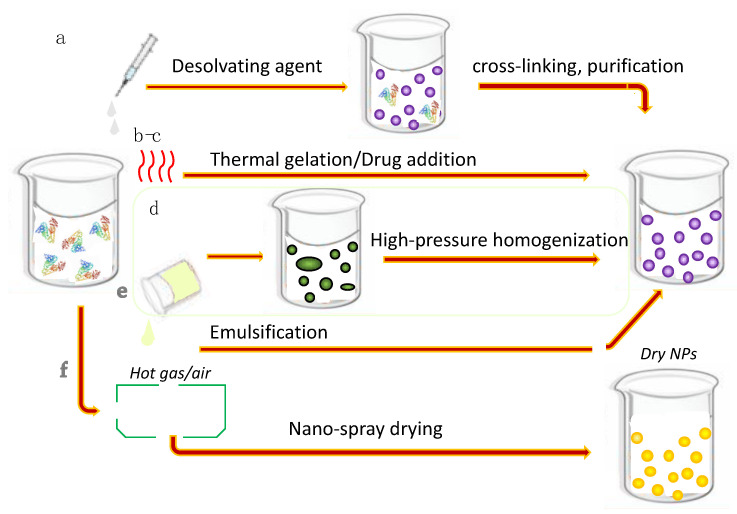
Schematic representation of the preparation methods for SANPs: (**a**) desolvation method, (**b**) thermal gelation, (**c**) self-assembly, (**d**) nab technology, (**e**) single emulsion method and (**f**) nanospray drying.

Several methods are possible to encapsulate the drugs in the NPs, including self-assembly [57], emulsification [58,59], thermal gelation, nanospray-drying and desolvation [60]. The techniques for the preparation of SANPs are summarized in Figure 3. Drug encapsulation renders several albumin moieties to form nano-sized objects, whereas with gelation, different precipitation techniques use protein denaturation to form the drug carriers. Emulsification, using nano- or mainly microemulsions with surfactant-stabilized droplets, is also widely used, resulting in NPs with average diameters of 100–1000 nm. Emulsification requires organic solvents, and the removal of both the surfactants and oily residues is problematic. However, both hydrophilic and hydrophobic drugs can be encapsulated by the process depending on whether the single- or double- emulsion route was applied. A wide array of drugs have been tried for drug delivery purposes. Among all these methods, desolvation is the most frequently used one, with ethanol used as a desolvating agent with the application of a cross-linker for stabilizing the NPs. One of the possibilities for hardening the particles via chemical cross-linking of the functional groups of amino acids is glutaric aldehyde [61]. The procedure was first introduced in 1978 by Marty et al. and was studied by Habeeb and Hiramoto [62,63]. It is still a commonly used method [64], however there are some obvious drawbacks such as the relatively long time it requires and the problems arising in biological systems. Another possibility is the application of *N*-(3-dimethylaminopropyl)-*N*-ethylcarbodiimide (EDC) by forming a peptide bond between the amino and carboxyl groups of amino acids by stirring for 6 h to ensure the cross-linking of all amino acid residues [65]. There are some papers discussing the application of different biodegradable reagents for chemical cross-linking [66]; for instance, Dextranox–MPEG cross-linking offers the possibility of steric stabilization [67]. Tazhbayev et al. applied citrate for the stabilization of hydroxyurea-loaded SANP for the purpose of both encapsulating the drug and ensuring hardening by biorelevant molecules [68]. The incubation of NPs offers the advantage of hardening, without the addition of further chemicals to the system; however, it has been mostly used in biphasic systems [69,70], Chen et al. described desolvatation in an acetone-water mixture [71]. The mechanism of thermal cross-linking is the condensation reaction between the carboxylic groups and amino groups of adjacent chains [72]. The latter is defined by an intraparticular interaction with the participation of mainly internal functional groups of the protein; thus, the functional groups of the surface remain unattached. The degree of the process is generally characterized by the amount of unreacted amino groups of the surface of the NPs, which can be determined by a 2, 4, 6-trinitrobenzenesulfonic acid (TNBS) reaction. This value is about 7–11 amino functional groups per HSA molecules in cases of cross-linking with glutaric aldehyde. The average particle diameter is usually independent of the amount of cross-linker or the pasteurization time and temperature, respectively. In general, it can be stated that the preparation method must be chosen according to the chemical character (e.g., solubility, interaction with albumin) of the applied drug and therapeutic target to ensure the required carrier properties. Moreover, a detailed examination of NP characteristics such as particle size and distribution, zeta potential, stability and in vitvo release must be carried out prior to in vivo studies.

## 4. Albumin-Based Products

The structure and role of albumins were first investigated some decades ago, yet, the use of albumin as a versatile drug carrier did not occur until decades later. There is some albumin-based formulations in clinical applications and some that are already on the market. The appearance and approval of Abraxane^®^ gave rise to further efforts to develop other albumin-based therapeutic products. Its success provides hope for the investigation of other clinically applicable systems. The composition applied in Abraxane^®^ is often an initial point of preparation. Due to the high accumulation of serum albumin in cancer tissues, there are other researches working towards the same goal. Moreover, cancer therapy requires a wide spectrum of usable pharmaceuticals for different types of cancer or for personalized therapy [73,74]. For treating diabetes, Levemir^®^ and Victoza^®^, myristic acid derivatives of human insulin or glucagon-like peptide 1 (GLP-1), act as long-acting peptides by binding to the fatty acid binding sites on circulating albumin in order to control glucose levels. The present albumin-based therapies are summarized in Table 1, presenting the name, the type, and the present stage of the clinical application with the proposed therapeutic use. In the near future, it is highly likely that there will be a new generation of albumin-based drugs that improve their bioavailability as well as albumin-based tailor-made products that exploit their enhanced permeation and retention (EPR) effects for reduced side effects and improved efficacy [75].

## 5. Future Perspective of Albumin Nanoparticles

There is a general difference between the application of nanomedicines to “conventional” medicinal products as the former are sophisticated and complex relative to the latter. There are particular properties that have raised important challenges for the industry and regulatory agencies as there has been a general lack of specific protocols to characterize these NPs [83,84]. The application of albumin-binding products such as Albumod^®^ (Affibody) or albumin-binding antibodies like Albud^®^ (GSK) and single-domain nanobodies such as Ablynx^®^ conjugating to drugs seems to be a very promising and exciting development [85]. Moreover, the albumins with tunable engagement with recycled FcRn allow the fine-tuning of therapeutic profiles. There is a need for the development of new transgenic humanized murine models to study the pharmacokinetics of these alternatives. Successful intracellular delivery has already been demonstrated for the albumin-based delivery of cancer drugs, but it is still unclear whether it is by a passive or an active targeted process and what the importance of the EPR effect or cellular receptors are [86]. It seems our knowledge on the intracellular pathways and nutrient release of HSA is limited. Moreover, to maximize the effect of albumin-based DDS and the targeted strategy for its delivery, a deeper understanding of NPs and cells should be achieved. For example, Al-Nakashly et al. observed that the geometry of cells can influence the nanoparticle uptake when poly(N-(2-hydroxypropyl)methacrylamide)-based micelles were investigated at a single-cell level on human (among others) carcinoma MCF-7 [87]. Similar experiments may shed a light on the design of functional NPs and the influence of geometrical traits of cells such as cell spreading area and cell shape on the uptake of NPs. [30,47] There is some basic, technical scientific research aspects that should be handled prior to moving forward to the next step. For instance, the effects of synthesis conditions (such as particles size and stability and special properties of SANPs) on the applicability of NPs in general should be regarded. Probing the various interface relationships between structure and activity determined by properties such as size and shape, surface properties such as roughness, and the presence of surface functional groups and their properties can be used to investigate engineered nanomaterials [37]. The net negative charge of albumin at physiological conditions could limit the efficacy of albumin as a drug carrier; however, this could be improved via modifications with functional groups such as carboxyl, hydroxyl or amino groups.

The combination of SANPs with other NPs, such as magnetic nanoparticles (MNPs), is also a promising strategy, where the albumin nanospheres wrap the MNPs and, thus, enhance the biocompatibility of MNPs and possess better control of release genes. This is partly because albumin nanospheres can be triggered by magnetic hyperthermia [88]. Moreover, the NPs can accumulate on the site of action through magnetic targeting, as presented by Zhang et al., who employed iron oxide superparamagnetic iron-oxide nanoparticles (SPIONs) encapsulated in albumin to deliver short hairpin RNA (shRNA). The system was precisely delivered to the tumor cells in the lung by placing a magnet close to the tumor by magnetic targeting [89]. Immunotherapy against cancer that is mediated by nucleic acids has enormous potential, as highlighted by recent developments, such as chimeric antigen receptors (CARs), to treat leukemia. Moreover, some study results show that the SANPs exclusively present promising properties for overcoming cancer drug resistance [90]. The appropriate tuning of drug release could be a potential strategy to overcome drug resistance. For instance, elucidating the mechanism of resistance by certain biomarkers may enhance albumin-based drug delivery based on the specific characteristics of the tumor and personalized therapy. However, such systems can be further improved by nanocarriers such as those based on albumin.

## 6. Conclusions

The albumin-based technology in the investigation of new, modern pharmaceuticals has been and continues to be a focus of interest for researchers. However, despite numerous efforts, only a few products have proceeded to clinical trial or application. The most obvious and probably the most successful utilization of this protein as a drug delivery system can be found in cancer therapy. However, at present, a serum albumin-based delivery solution has been developed for diabetes and HIV. To improve the effectiveness of albumin-based therapeutic agents, researchers should focus on developing a better understanding of and communication between preparation conditions and the aim of therapeutic utilization in order to develop improved, modern target-specific applications and smart formulations, and to improve high-scale production possibilities. However, the application of “green” and sustainable sources is improving, and there is an increasing interdisciplinary scientific approach to working towards multifunctional, new-generation DDS. Moreover, there is more of a drive towards the application of the considerable knowledge gained from different scientific communities and specific areas of research. Unquestionable possibilities have emerged in the presence of r-albumin and, hopefully, further clinically-approved products, advanced treatments and personal therapies for leading (mostly human) diseases will be developed.

## Figures and Tables

**Table 1:** A summary of clinically approved albumin-related formulations.

Name	Type	Clinical Approval Stage	Clinical Application	Refs.
Abraxane (ABI-007)	Paclitaxel HSA-bound NP	Approved	Metastatic breast cancer, non-small cell lung cancer	[75]
ABI-008	Doxetaxel-albumin NP	Phase II	Cancer	[76]
ABI-009	Rapamycin-albumin NP	Phase I	Cancer	[77]
Optison	Perflutren protein-type A microsphere injectable suspension	Approved	Contrast agentfor ultrasound imaging	[78]
Nanocoll	^99m^Tc-labelled HSA	Approved	SPECT scan for sentinel node localization in breast cancer	[79]
Levemir	Fatty acid–insulin conjugate	Approved	Diabetes	[80]
Liraglutide	Fatty acid–peptide conjugate	Approved	Diabetes	[80]
Albiglutide	Peptide–HSA conjugate	Approved	Diabetes	[81]
Aldoxorubicin	Doxorubicin–maleimide conjugate	Phase III	Soft tissue sarcomas, small cell lung cancer	[80]
Albinterferon	Interferon alpha (IFN-)–HSA conjugate	Phase III	Hepatitis C	[79]
MTX–HSA	Methotrexate–HSA conjugate	Phase II	Metastatic translational cell cancer	[82]
Abliglutide	Peptide–HSA conjugate	Approved	Diabetes mellitus, Type 2	[76]
